# Inkjet Printing of Cadmium-Free Quantum Dots-Based Electroluminescent Devices

**DOI:** 10.1021/acsami.5c01588

**Published:** 2025-04-03

**Authors:** Min Fu, Juan José Santaella, Stephen D. Evans, Kevin Critchley

**Affiliations:** † School of Physics and Astronomy, University of Leeds, Leeds LS2 9JT, U.K.; ‡ Department of Electronics, VALEO Lighting Systems, Martos 23600, Spain

**Keywords:** inkjet printing, quantum dots, quantum dot-based light-emitting diodes, cadmium free, coffee ring effect

## Abstract

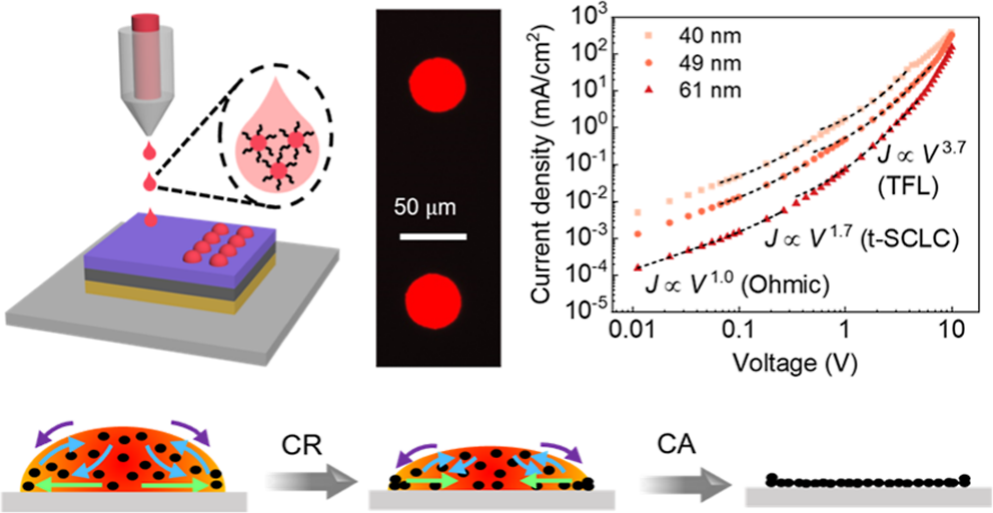

InP quantum dots (QDs) have excellent optoelectronic properties and less toxicity than Cd-based QDs, making them excellent candidates for QD-based light-emitting diodes (QLEDs). Inkjet printing is a promising technology to replace other methods, such as spin coating, vacuum evaporation, and lithography, for assembling lower-cost and high-resolution QLEDs. However, inkjet printing faces the challenge of a coffee ring effect. To address this, we combined the solutal and thermal Marangoni effects by employing a binary solvent system (cyclohexylbenzene and decane) and heating the substrate during printing. The thermal Marangoni effect, which has been underexplored in previous studies of inkjet-printed QLEDs, is a focal point of this work. Uniform patterns were obtained with a volume ratio of 20% decane and a substrate temperature of 60 °C. The evaporation of the solvents from QD ink droplets behaved differently at different substrate temperatures, i.e., stick-jump mode at 20 and 40 °C and stick-slide mode at 60 °C. Consequently, the inkjet-printed InP QLEDs without the coffee ring effect were successfully assembled. Furthermore, increasing the electron transport layer (ETL) thickness reduced trap density when it was exposed to the air and prevented the deterioration of the QD layer from water vapor and oxygen exposure. This is likely due to the decrease in oxygen vacancies in the ETL, mitigating the defect-dependent exciton quenching at the ETL/QD interface.

## Introduction

Quantum dot (QD)-based light-emitting diodes (QLEDs) are regarded as a likely next-generation display technology owing to their high color purity, low power consumption, and ultrahigh contrast.^1^ Yet, manufacturing high-resolution, large-scale, and low-cost QLEDs remains challenging. Spin coating (SC) is a widely used solution-processed method because it is well-established for lithographic techniques and enables the wet deposition of smooth and uniform thin films rapidly.^2^ However, SC has the disadvantage that a majority of the material being coated is lost as waste. Vacuum evaporation is commonly used to manufacture large-scale organic LEDs, but some colloidal QDs cannot be sublimated or evaporated.^3^ The remaining materials deposited on the mask are wasted, and this method must be performed under high-vacuum conditions. Lithography can precisely pattern high-resolution QD pixels through selective illumination.^4^ However, exposure to UV light and the use of harsh chemicals during the process can degrade the optical properties of the QDs. In contrast, inkjet printing (IJP) technology uses fewer materials, creates patterns without masks, and achieves high resolution, making it a promising candidate for industrial QLED assembly.^5^ Despite its advantages, IJP is challenged by the coffee ring effect (CRE), where capillary flow, driven by a faster evaporation rate at the droplet’s edge, causes liquid from the interior to move outward.^6^ This movement transports solutes to the fixed contact line, forming a ring of the deposited material at the contact line.

Several methods have been proposed to mitigate the CRE, including enhancing the Marangoni effect (ME),^7^ engineering the substrate surface with two-dimensional or three-dimensional structures,^8^ and electrowetting.^9^ ME involves the transport of solvents from regions of lower surface tension to those with higher surface tension and, therefore, will transport solutes from the edge back into the interior of the droplet. There are two types of Marangoni flows. One is the concentration-driven Marangoni flow achieved by introducing an additional solvent or surfactant.^10^ For a binary solvent system, as the droplet evaporates, a difference in surface tension between the edge and the center generates an inward Marangoni flow, balancing the outward capillary flow. The second type of Marangoni flow, which is thermally driven and exhibits a circulating motion, has been less studied in previous reports. The initial flow direction depends on the thermal conductivity ratio (*K*_R_ = *K*_s_/*K*_l_) between the substrate (*K*_s_) and the liquid (*K*_l_).^11^ If *K*_R_ is greater than 2, indicating an efficient conductor substrate, heat transfers from the contact line to the droplet center because it is warmest at the contact line. Conversely, when *K*_R_ is less than 1.45, the flow reverses due to the highest evaporation rate at the contact line, and the droplet temperature cannot be maintained without sufficient energy. For 1.45 < *K*_R_ < 2, the Marangoni flow direction depends on the critical contact angle. To balance the capillary flow with the Marangoni flow, it is crucial to optimize the ink formulation, particularly the selection of solvents based on their rheological properties, boiling points, thermal conductivity, and compatibility.

Heavy metals such as Cd, Pb, and Hg have been restricted in many electronics in the EU since 2011 due to their intrinsic toxicity.^12^ Therefore, the development of Cd/Pb/Hg-free QD-based QLEDs has become a key focus for commercial applications. Some alternative QDs have been developed to replace Cd-based QDs such as lead-free perovskite QDs,^13^ CuInS_2_ QDs,^14^ and InP QDs.^15^ Compared with CdSe QDs, InP QDs have a slightly smaller bulk band gap (1.34 eV vs 1.74 eV), much larger exciton Bohr radius (10 nm vs 3 nm), and reduced toxicity, showing great potential for wider technological use, for example, QLEDs.^16^ Currently, the optical properties (e.g., PLQY and emission line width) of state-of-the-art InP QDs are comparable to those of CdSe QDs.^17,18^ However, most inkjet-printed QLEDs are based on CdSe QDs and Pb-based perovskite QDs, with a record EQE of 23.1% and 14.3%, respectively.^18,19^ The inkjet-printed red, green, and blue InP QLEDs have only appeared in the past three years, achieving the highest EQE of 8.1%, 0.7%, and 0.15%, respectively,^20−22^ which are much lower than that of spin-coated analogues, i.e., 23.5%,^17^ 26.7%,^23^ and 2.6%.^24^ This results from the increased vulnerability of In and P to air and the difference in energy band alignments.^25^ The higher covalency of InP has hindered synthetic advancements, as it requires highly reactive precursors for lattice formation, making the resulting nanocrystals more susceptible to lattice defects. Therefore, shell engineering and optimizing the QD ink formulation and device structure have been proposed to enhance the electrical performance of inkjet-printed InP QLEDs.^20−22^ Additionally, it was reported that the electrical performance of QLEDs deteriorates when the electron transport layer (ETL, Zn_1–*x*_Mg_*x*_O) is exposed to air.^26^ This may be attributed to the increase of trap density (density of carriers occupying trap states) in the ETL, especially oxygen vacancies, resulting in exciton quenching at the QD/ETL interface.^27^

This study aims to fabricate red-emitting InP QLEDs by IJP the QD layer without the CRE. Cyclohexylbenzene (CHB) and decane were selected to formulate the QD inks with different volume percentages (vol %), and we investigated the impact of the vol % of decane and substrate temperature (*T*_sub_) on the CRE. The study focuses on the thermal Marangoni effect to overcome the formation of coffee rings. To further study how coffee rings formed at different *T*_sub_, real-time evaporation of QD inks was observed from both top and side views. The electrical properties of InP QLEDs with different ETL thicknesses have been investigated to mitigate the deterioration.

## Results and Discussion

### InP/ZnSe_*x*_S_1–*x*_/ZnS QDs

**Figure 1:**
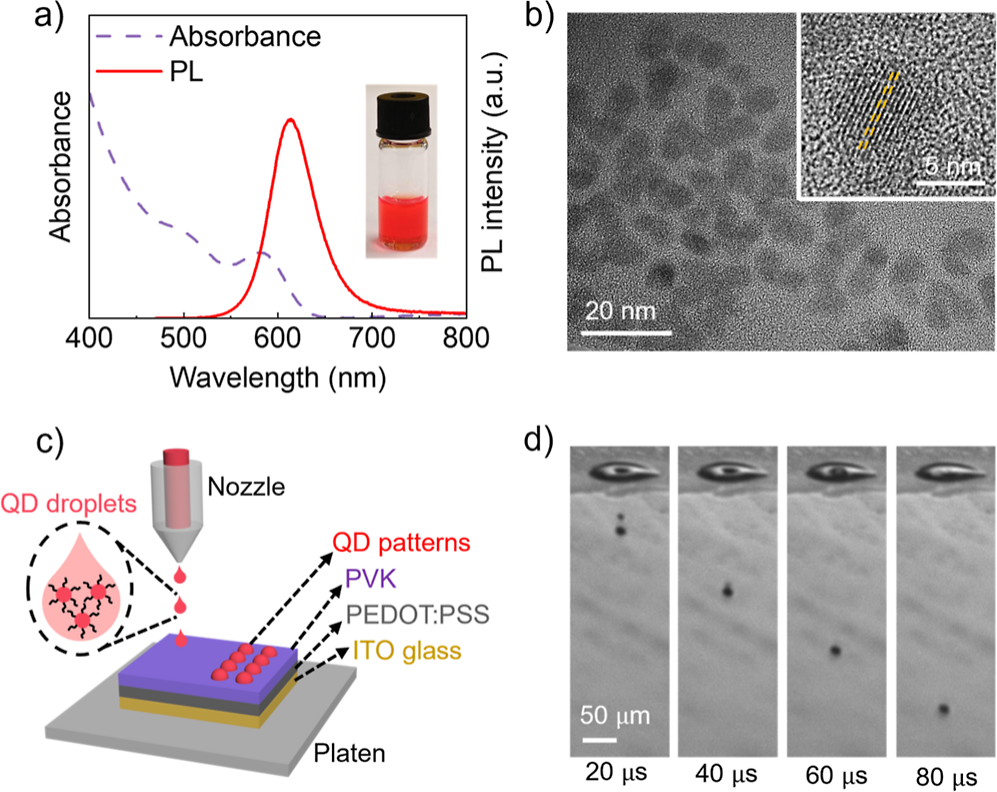
Inkjet printing of InP/ZnSe_*x*_S_1–*x*_/ZnS QD inks. (a) Absorption and PL spectra of ink-20. The inset is a photograph of the QD ink. (b) High-resolution TEM images of QDs. (c) A schematic diagram of inkjet printing QD inks. (d) The evolution of a single droplet over time, following ejection from the nozzle.

The synthesis of colloidal multishelled InP/ZnSe_*x*_S_1–*x*_/ZnS QDs was slightly modified from the previously reported hot-injection method^28^ and is described in the Experimental Section. After the double shell (ZnSe_*x*_S_1–*x*_/ZnS) was grown onto the InP cores, the first excitation peak red-shifted to 581 nm, corresponding to a reduced band gap of 2.01 eV and a small Stokes’ shift of 33 nm (Figure 1a). The optical properties of the InP core and InP/ZnSe_*x*_S_1–*x*_ are shown in Figure S1a–c. The InP/ZnSe_*x*_S_1–*x*_/ZnS QD dispersion had a distinctive reddish-orange color in ambient lighting conditions (Figure 1a inset). The PL emission spectrum from the dispersion showed a single peak with a maximum at 614 nm and a full width at half-maximum of 56 nm which is comparable to some reported values but is inferior to that of state-of-the-art InP QDs (<40 nm).^17,29^ The broader emission than Cd-based QDs associated with structural and electronic disorders.^29,30^ The peak was slightly asymmetric, with a slight tail into the red region due to an asymmetric size distribution of the QDs (e.g., more larger QDs than smaller QDs) and is common, particularly for the larger QDs.^31^ The PLQY of InP/ZnSe_*x*_S_1–*x*_ and InP/ZnSe_*x*_S_1–*x*_/ZnS QDs was determined to be 58 ± 1% and 81 ± 2%, respectively, suggesting that the multiple shells were effective in protecting and removing the surface trap states from the core–shell interface (Table S1). The composition-gradient ZnSe_*x*_S_1–*x*_ intermediate shell reportedly relieves interfacial compressive strain at the InP/ZnS interface, contributing to the higher PLQY.^32^Figure S1d shows the TRPL curves of QDs, and the decay times of components of QDs are summarized in Table S2. The corresponding analysis is described in detail in the Supporting Information. The average lifetime (τ_avg_) and amplitudes increased after coating the shell, but QDs with a single shell and double shell did not show big changes, suggesting a thin outmost shell.

TEM images of QDs showed that the nanocrystals were irregular in shape (Figure 1b), with an average size of 8.1 ± 0.7 nm (measured as the longest distance, Figure S2), and an interplanar spacing of 3.37 Å corresponds to the cubic crystalline plane of (111) (Figure 1b inset). The radius of the InP core was estimated to be 1.8 nm by the previously reported equation,^33^ thus obtaining the total shell thickness of 2.3 nm (c.a. 4.3 monolayers, calculated based on the lattice constant of ZnS −0.541 nm^34^). The relatively thick shell can enhance stability and suppress nonradiative Forster resonant energy transfer in InP/ZnSe_*x*_S_1–*x*_/ZnS core/shell QDs, contributing to more stable and efficient QLEDs.^27^

### Ink Formulations and Stability

The QD inks that meet the criteria for inkjet printing were formulated as described below. The solvents CHB and decane have boiling points of 239 and 174 °C, respectively, and were both found suitable to disperse the octanethiol-stabilized QDs. The QD inks with 3 vol %, 10 vol %, 20 vol %, and 80 vol % of decane were denoted as ink-3, ink-10, ink-20, and ink-80, respectively. The vol % of decane was selected based on the QD ink formulation reported by Li et al., where CHB was the primary solvent.^35^ They noted that the CHB/decane solvent system was ineffective on the PVK film. However, it can be used by leveraging the thermal Marangoni effect in this work, which is discussed later. The inks were stable as stored in a glovebox, exhibiting nearly the same PLQY and emission peak after 10 days (Figure S3). The printability of the QD inks can be determined by calculating the figure of merit, *Z*,^36^ given by
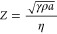
1where η, ρ, γ, and *a* represent the viscosity, density, surface tension of the ink, and the nozzle diameter, respectively. Table 1 gives a summary of the relevant properties of four QD inks, and the characterization of these parameters is described in the Experimental Section.

**Table 1:** Rheological Properties, Printability, and Solutal Marangoni Strength of QD Inks with Different Vol. % of Decane in CHB

QD inks	ink-3	ink-10	ink-20	ink-80
decane content (vol %)	3	10	20	80
viscosity (η, mPa s)	2.80 ± 0.01	2.44 ± 0.01	2.14 ± 0.01	1.12 ± 0.01
surface tension (γ, mN m^–1^)	31.5 ± 0.6	30.7 ± 0.1	30.2 ± 0.1	26.1 ± 0.1
droplet contact angle (deg)	15.3 ± 0.6	12.3 ± 1.7	11.5 ± 1.3	10.8 ± 0.9
density (ρ, g cm^–3^)	0.93 ± 0.01	0.93 ± 0.01	0.92 ± 0.01	0.77 ± 0.01
*Z* value	6.7	7.7	8.6	13.8
Ma_s_ strength (Δγ/η)	0.29	0.66	0.98	5.54

It was reported that for good printability the ink’s *Z*-number should fall into the range between 1 and 10,^37^ and all except ink-80 fall within this range. ink-80 was also printed successfully despite its *Z* value > 10, which means it is a sufficient but not necessary condition. The low contact angle (<16°) of the CHB/decane mixture on the PVK film demonstrates the high surface energy and excellent surface wettability of PVK.^38^ Reducing the contact angle increases the contact area between the droplet and the solid surface while decreasing the droplet’s thickness.^39^ This enhances the heat conduction through the droplet, accelerating its evaporation rate. If evaporation occurs significantly faster than particle movement, the formation of a coffee ring may be suppressed.^40^ In this study, PVK was chosen as the hole transport layer rather than another commonly used TFB because PVK has better resistance to CHB and decane. The mean thickness of PVK films barely changed after rinsing with the ink solvents, while it significantly decreased for TFB films (Figure S4). These inks were printed on a PVK-coated glass substrate (Figure 1c). Before printing, a few parameters were examined to prevent satellites and to form solid films, including voltage and drop spacing. The clear spherical droplet was ejected without satellites and off-axis drops under a printing voltage of 9.5 V, corresponding to a velocity of ∼4.3 m/s (Figure 1d). Jetting at higher voltages led to faster drops and bigger drops, but the satellite was generated when the voltage was greater than 10.0 V (Figure S5). Voids appeared when the drop spacing (the distance between two neighboring droplets) > 20 μm, so the maximum drop spacing of 20 μm was set to meet the requirement of forming continuous patterns (Figure S6).

### Effect of the Vol % of Decane and *T*_sub_ on the Coffee Ring Effect

**Figure 2:**
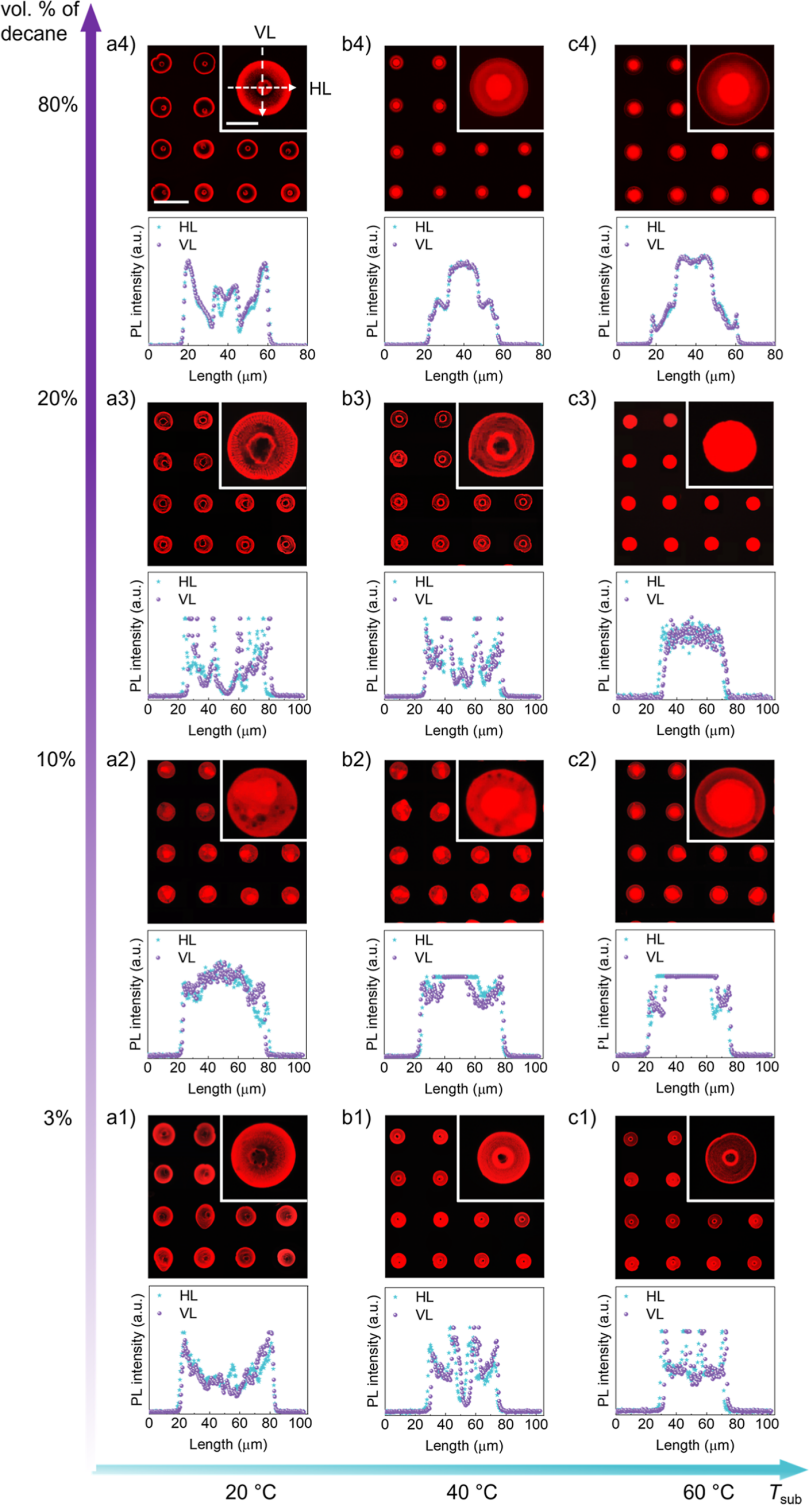
PL images and corresponding line profiles of QD patterns by printing the QD inks with 3 vol %, 10 vol %, 20 vol %, and 80 vol % of decane on the PVK-coated glass substrate at the *T*_sub_ of (a1–a4) 20 °C, (b1–b4) 40 °C, and (c1–c4) 60 °C. Each PL image has an insert at the top right to enlarge the spot. The droplet volume is 25 ± 5 pL. The scale bars for the spot arrays and the inset spots are 100 and 25 μm, respectively. HL and VL indicate horizontal and vertical lines, respectively, passing through the center of the inset spot.

The QD inks were printed on the PVK film at different substrate temperatures, *T*_sub_, of 20 °C, 40 °C, and 60 °C using the above printing parameters. The fluorescent images and corresponding line profiles of the printed circular patterns are shown in Figure 2. The droplets exhibited multiple coffee rings after evaporation except ink-20 printed at the *T*_sub_ of 60 °C, indicating multiple times of pinning of the contact line. At the *T*_sub_ of 20 °C, all ink droplets apart from ink-10 showed two coffee rings and a dark center due to the prolonged drying time and the longer pinning period (Figure 2a1,a3, and a4). The nonuniformity of the ink-10 droplets at the *T*_sub_ of both 20 and 40 °C can be attributed to the undesirable dissolvability and aggregation during solvent evaporation (Figure 2a2,b2).^35^ As the *T*_sub_ increased from 20 to 40 °C, the number of coffee rings remained the same, but the outermost coffee ring narrowed because the extra thermal energy shortened the contact line pinning time at the edge (Figure 2b1,b3). A small bright center reveals that the contact line of ink-80 droplets was depinned during the final drying stage (Figure 2b4). When the *T*_sub_ reached 60 °C, the ink-3 droplets showed the same CRE as at the *T*_sub_ of both 20 and 40 °C because the small decane volume evaporated rapidly initially, leaving a CHB-only solvent system (Figure 2c1). Due to the very short pinning time, only a weak coffee ring at the edge and a larger bright center were observed for the ink-10 and ink-80 droplets (Figure 2c2,c4). Yet, uniform patterns were achieved when ink-20 was printed at 60 °C (Figure 2c3).

In terms of the motion of capillary flow (Ca) and Marangoni flow during drying, the contact line was pinned after the droplets were deposited, and the evaporation of decane at the edge created the outward Ca. As the initial homogeneous binary components changed, spatial variations in composition emerged, leading to corresponding interfacial γ gradient and a solutal Marangoni flow (Ma_s_) along the interface from the apex to the edge of the droplet. The strength of the Ma_s_ was reported to be proportional to the ratio of interfacial γ gradient (Δγ = γ_CHB_ – γ_ink_) and viscosity, Ma_s_ ∝ Δγ/η,^41,42^ where γ_CHB_ is γ of the QD ink with 100 vol % of CHB (32.3 mN/m). It increases by increasing the vol % of decane (see Table 1) and moves from the center to the edge. If Ca and Ma_s_ are too strong, then the CRE will be the resultant pattern. To overcome the outward flows, the strength of the thermal Marangoni flow (Ma_T_) must be enhanced by increasing the *T*_sub_. The initial flow direction depends on the ratio of thermal conductivity between the glass (*K*_s_ = 1.05) and the CHB/decane mixture (*K*_l_ = ∼0.13) at RT, which is 8.1. As this ratio is > 2, thermal energy transferred to the droplet caused the highest temperature at the edge, where the conduction pathway was minimized. In contrast, the droplet apex experienced a lower temperature due to the longer conduction pathway. Therefore, the circulation flow caused by Ma_T_ spatially moved inward and then outward. We speculated that the capillary and net Marangoni flow reached equilibrium for ink-20 when *T*_sub_ = 60 °C.

### Evaporation Modes of the Ink Droplets

**Figure 3:**
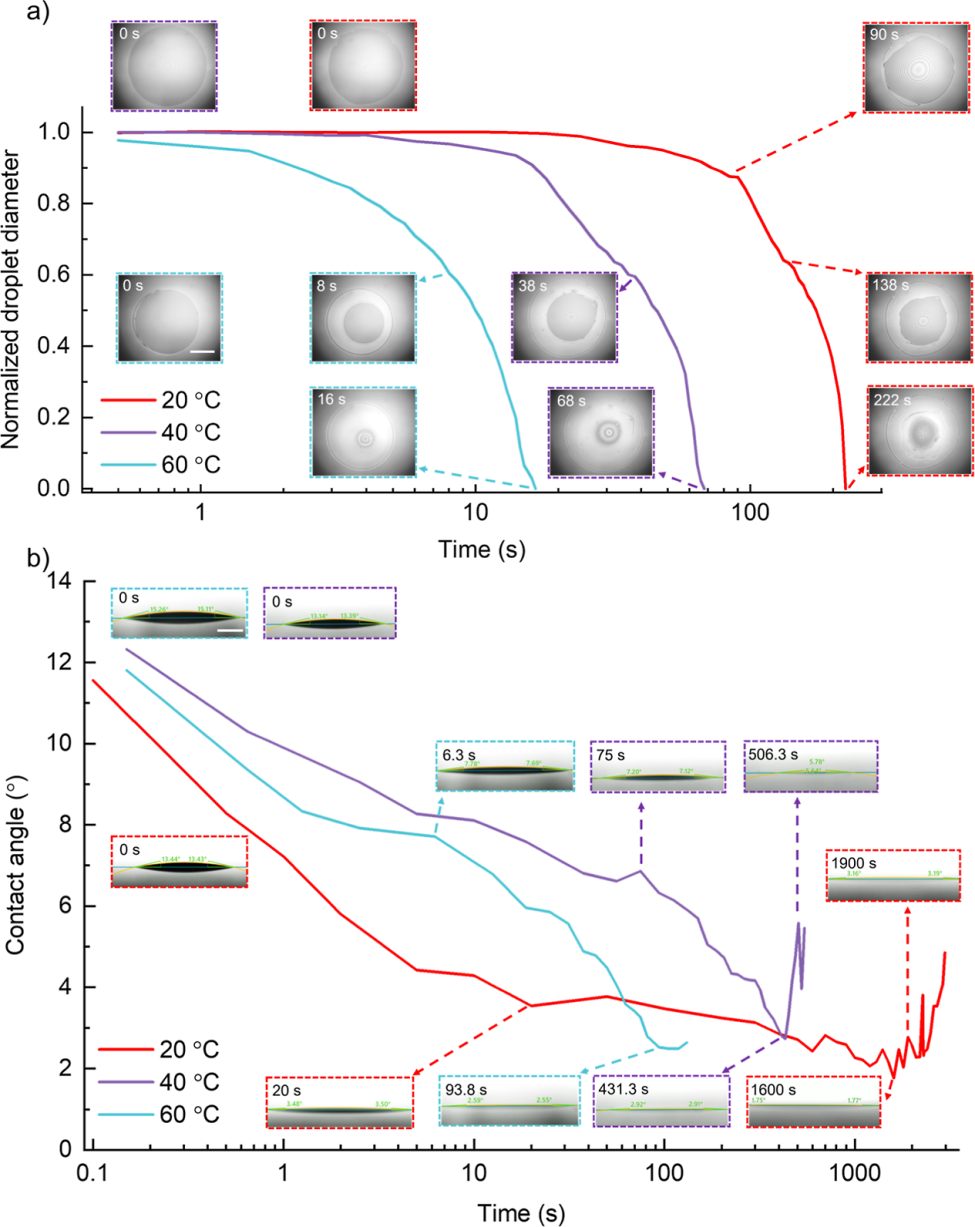
Evolution of (a) droplet diameter and (b) contact angle of ink-20 drying on the PVK-coated glass substrate at the *T*_sub_ of 20 °C, 40 °C, and 60 °C. The scale bars in (a,b) are 200 and 300 μm, respectively. The droplet volumes in (a,b) are 46 ± 10 nL and 310 ± 70 nL.

The real-time evaporation of the ink-20 droplets on the PVK film at the *T*_sub_ of 20 °C, 40 °C, and 60 °C was observed from both top and side views to study how coffee rings formed through the evolution of droplet diameter and contact angle (Figure 3). Notably, the droplet size and volume in Figure 2 were smaller than those in Figure 3 because different instruments with varying capabilities were used, resulting in different time scales. For droplets with different sizes, the evaporation, pinning, and depinning times vary during evaporation, but the trends in droplet diameter and contact angle evolution remain consistent.^43^ Initially, a plateau in droplet diameter indicated that the contact line was pinned while the contact angle was reduced, forming the outermost coffee ring, a phase which is known as the constant radius (CR) mode.^44^ The pinning time decreased with increasing *T*_sub_ (<1 s at 60 °C, Figure 3a). Subsequently, the contact line receded, and the contact angle partially recovered at 20 and 40 °C but plateaued at 60 °C (at ∼94 s, Figure 3b) until drying was complete, a phase termed the constant angle (CA) mode.^45^ Then, the droplet diameter plateaued again at 38 s, with the contact angle partially recovering several times at 40 °C, i.e., alternating the CR and CA modes until the contact line receded to zero, forming more coffee rings. At 20 °C, the droplet behaved the same as at 40 °C but repeated more cycles in the CR and CA modes. Thus, the ink-20 droplets evolved through a mixed drying mode instead of a single CR or CA mode.

**Figure 4:**
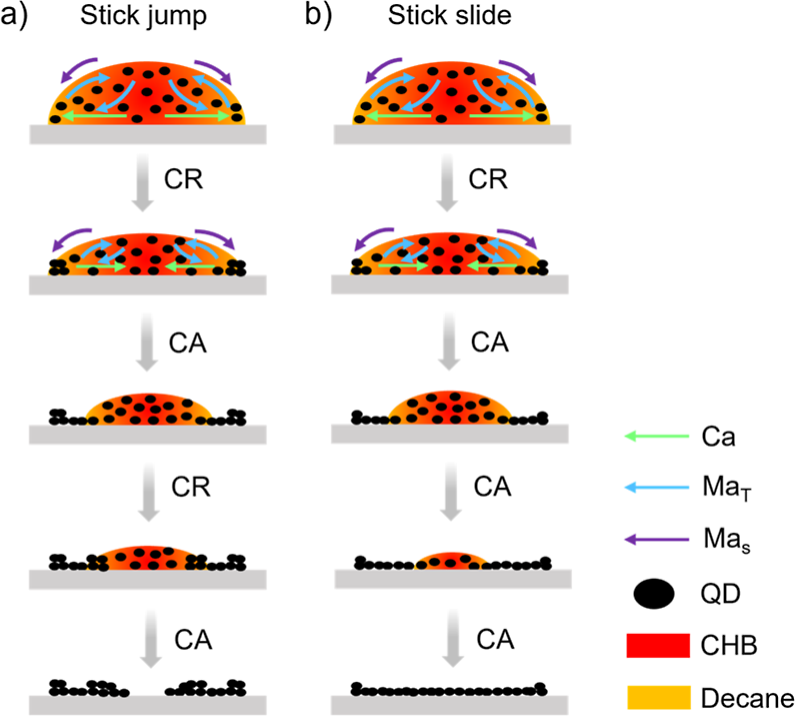
Schematics of the evaporation modes of QD droplets drying on the PVK film at different *T*_sub_. (a) SJ mode at 20 and 40 °C. (b) SS mode at 60 °C.

At the *T*_sub_ of 20 and 40 °C, the combined effects of Ca and Ma_s_ initially drive liquid from the center to the periphery, reducing the contact angle while keeping the contact line pinned in a CR. This transport of QDs to the edge forms an outermost coffee ring. Subsequently, the Ma_T_ and Ca restore the droplet’s original shape, triggering the contact line to recede. The process then was repeated multiple times until the droplet fully evaporates. This evolution is called the stick-jump (SJ) mode (Figure 4a),^46^ where multiple coffee rings originated from the CR mode. In contrast, when drying at 60 °C, the initial drying phase in the CR mode follows the same pattern, but due to the stronger strength of Ma_T_ and rapid evaporation, its duration is significantly shorter. The drying process then transitions into the CA mode, continuing until the contact line fully recedes. This evolution belongs to another mixed mode, starting with a CR mode followed by a CA mode until the evaporation was completed, known as the stick-slide (SS) mode (Figure 4b).^47^

### Electrical Performance of InP QLEDs

**Figure 5:**
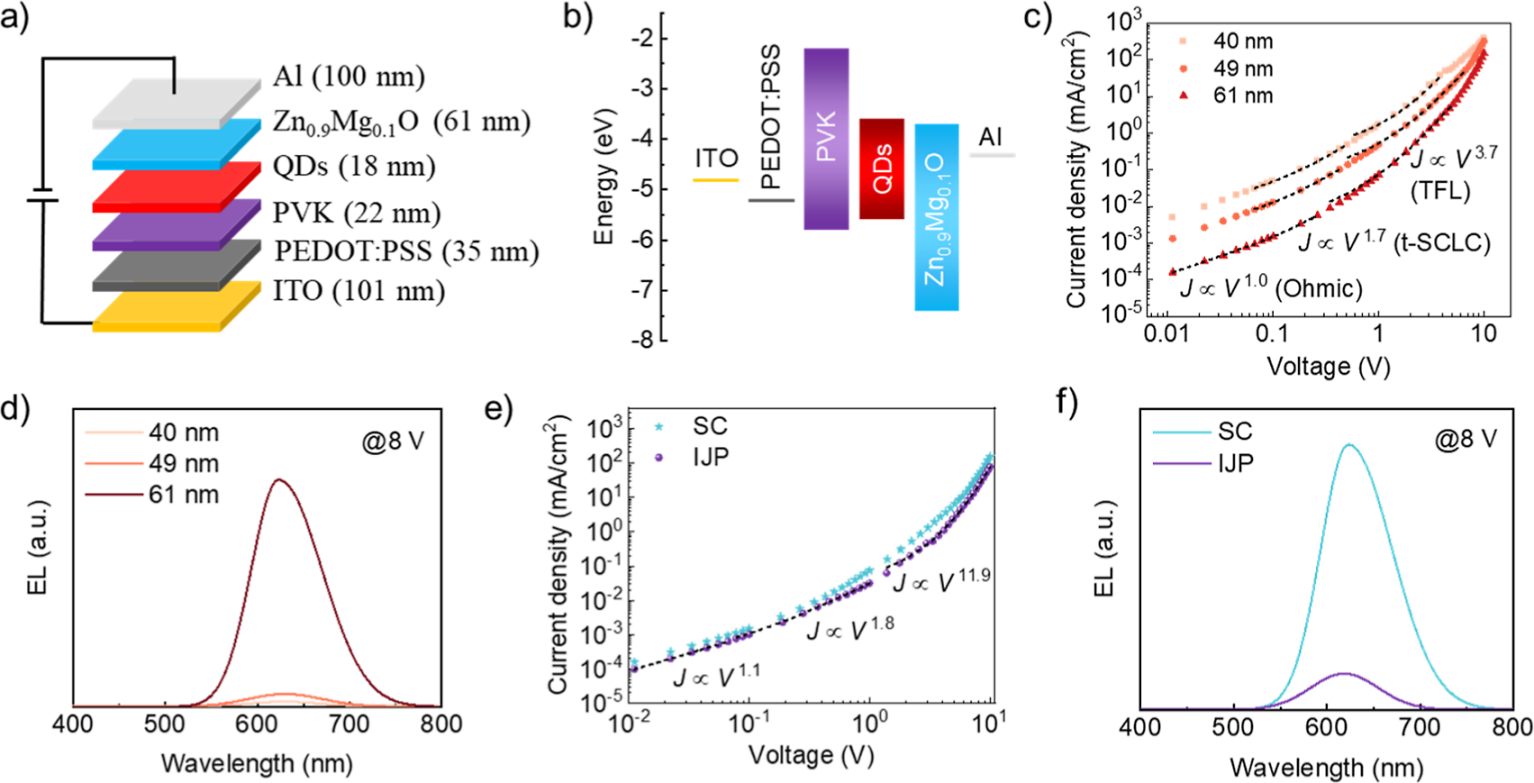
Electrical performance of inkjet-printed InP QLEDs. (a) The device structure and (b) the energy band diagram of QLEDs. (c) *J*–*V* behavior and (d) EL spectra (at 8 V) of QLEDs with different thicknesses of the ETL. (e) *J*–*V* behavior and (f) EL spectra (at 8 V) of spin-coated and inkjet-printed QLEDs with the same ETL thickness of 61 nm. The black dashed lines in (c,e) are fitting curves of *J* ∼ *V*^*n*^.

The structure of inkjet-printed QLEDs consists of indium tin oxide (ITO, 101 nm), poly(ethylenedioxythiophene):polystyrenesulfonate (PEDOT:PSS, 35 nm), PVK (22 nm), InP/ZnSe_*x*_S_1–*x*_/ZnS (QDs, 18 nm), Zn_0.9_Mg_0.1_O NPs (61 nm), and Al (100 nm), as shown in Figure 5a. Adding a small amount of alcohol into the PEDOT:PSS ink was reported to enhance its conductivity due to its clean-off effect on the insulting PSS.^48^ In this work, the addition of 20 vol % of IPA also led to a lower contact angle on the ITO glass (Figure S7), facilitating the formation of smoother and more uniform PEDOT:PSS films. The QD layer was either spin-coated or inkjet-printed (Scheme S1). The thicknesses of these spin-coated and inkjet-printed functional layers are summarized in Table S3. Their AFM images exhibited smooth and pinhole-free features (Figure S8). The average thickness of one printing layer of the QD film was about 18 nm (Figure S9), and the roughness (*R*_ms_, 2.9 nm) was comparable to that of the spin-coated one (2.4 nm). Figure 5b shows the corresponding energy band alignment of the functional materials in the devices. Doping Mg into ZnO NPs has been reported to reduce electron mobility and elevate the conduction band minimum which is beneficial for a more balanced charge injection, and the optimal doping molar ratio was reported to be 12.5%.^49^ The synthesis protocol of Zn_0.9_Mg_0.1_O NPs is described in the Experimental Section. To enhance the monodispersity of the Zn_0.9_Mg_0.1_O NPs, ethanolamine was added as the surfactant. The turbidity of the Zn_0.9_Mg_0.1_O-EA NP dispersion was visibly reduced in ambient lighting conditions (Figure S10a), and the roughness of Zn_0.9_Mg_0.1_O-EA NP films reduced from 5.6 to 2.6 nm (Figure S8f,g). The optical bandgap of 3.65 eV (Figure S10b) was comparable to previously reported results^50^ but higher than that of bulk ZnO (3.2–3.3 eV), indicating greater spatial confinement of photogenerated charge carriers in the smaller ZnO NPs.^51^ The synthesized Zn_0.9_Mg_0.1_O NPs showed an irregular shape and great crystallinity with an interplanar spacing of 2.73 Å, which corresponds to the crystalline plane of (110), see Figure S11a,b. The narrow size distribution of 3.9 ± 0.5 nm was similar to the reported results (Figure S11c).^50^ The atomic doping ratio of 10% Mg was determined by EDX (Figure S11d).

In our system, the thermal evaporator was not connected to the glovebox, so the ETL was inevitably exposed to air with a relative humidity (RH) of 55% ± 5% for approximately 20 min. During the transfer, oxygen changes the behavior of the charge transport in the ETL because it can trap electrons. Water vapor accumulates on the surface of the Zn_0.9_Mg_0.1_O film and soaks into the QD layer when RH > 60%, quenching the QD layer and deteriorating device performance.^26^ We assumed that thicker ETL (>40 nm) can alleviate the deterioration of the ETL and QD layer, so the electrical performance of QLEDs with different ETL thicknesses was investigated. The ETL thickness of 40 nm was commonly used in literature (without exposure to air),^52^ and resistivity was reported to increase for films thicker than 50 nm because more carriers were trapped at grain boundaries.^53^ Thus, thicknesses of 40, 49, and 61 nm were selected for this study. The *J*–*V* characteristics of spin-coated InP QLEDs with different thicknesses of Zn_0.9_Mg_0.1_O films suggested a *J* ∼ *V*^*n*^ relation (Figure 5c).^54^ They exhibited three different current regimes: ohmic current (*n* = 1), trap-limited space charge limited current (*t*-SCLC, *n* = 1 ∼ 2), and trap-filled limited current (TFL, *n* = 2 ∼ 100). The corresponding power exponents are shown in Table 2, and the bigger *n* in the TFL region indicates the presence of more trap states. The threshold voltage (*V*_TFL_, the voltage transits from the ohmic to the *t*-SCLC region) and trap density (*N*_t_) were reduced by half and two-thirds, respectively, when the ETL thickness increased from 40 to 61 nm. The trap density can be calculated by^27^
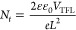
2

**Table 2:** Power Exponent, *V*_TFL_, and *N*_t_ of InP QLEDs with Different ETL Thicknesses

thickness of the ETL (nm)	ohmic	*nt*-SCLC	TFL	*V*_TFL_ (V)	*N*_t_ (*N*_0_)
40 (SC)	1.0	2.0	4.9	0.51	1.0
49 (SC)	1.0	2.0	3.8	0.43	0.56
61 (SC)	1.0	1.7	3.7	0.26	0.34
61 (IJP)	1.1	1.8	11.9	1.0	1.31

where ε, ε_0_, *e*, and *L* are the relative permittivity of Zn_0.9_Mg_0.1_O NPs, vacuum permittivity, elementary charge, and the thickness of Zn_0.9_Mg_0.1_O NP films, respectively. The EL intensity of InP QLEDs significantly improved due to the lower trap states (Figure 5d). The lower trap density is likely attributed to a reduction in oxygen vacancies within the ETL, which minimizes defect-related exciton quenching at the ETL/QD interface.^55^ The inkjet-printed devices showed higher *V*_TFL_, *N*_t_, and *n* in the TFL region than the spin-coated devices, which is likely due to the increasing air exposure time of the QD layer being printed in an ambient atmosphere. This introduces surface oxides and more trap states in the QD layer, thus impacting the QD-ETL interfacial electron transport (Figure 5e).^55,56^ It could also account for potential contributions from other layers within the device. The PVK film could be slightly corroded by the ink solvents and was exposed to air for a short time before printing the QD layer (Figure S4b). Water and oxygen might physiosorb on the PVK film, reducing the hole injection. The luminance of InP QD-LEDs increased with increasing applied voltage, but it reached a plateau (620 and 250 cd m^–2^) at approximately 10.0 and 11.5 V for the spin-coated and inkjet-printed devices, respectively, likely due to the field-induced quenching^57^ (Figure S12a). The maximum EQE values of spin-coated and inkjet-printed ones were 0.5% and 0.2%, respectively (Figure S12b). The EL intensity of inkjet-printed devices was only one-seventh of that of spin-coated devices (Figures 5f and S13), but they exhibited comparable EL peak maxima (620 nm vs 623 nm). Compared with the PL, the EL spectrum also exhibits asymmetry and a red shift, which can also be attributed to the combination of QD size variation, but additionally, the electric field-accompanying Stark effect, and the inter-QD Forster resonant energy transfer (due to close proximity in the film).^58,59^ Apart from the undesirable compatibility of TFB with the ink solvents, the disadvantage of TFB-based InP QLEDs also included a parasitic emission centered at 436 nm because the deep-lying lowest unoccupied molecular orbit of TFB led to the flow of electrons from the QD layer to the TFB layer (Figure S14).^28^

**Figure 6:**
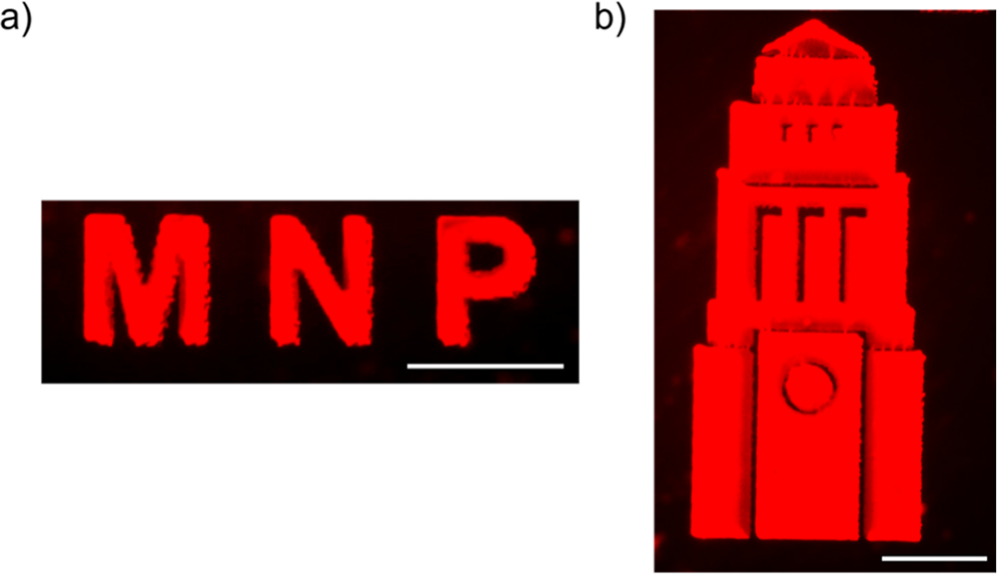
PL images of an inkjet-printed (a) “MNP” letters and (b) Leeds University icon by printing ink-20 on the PVK-coated glass substrate at the *T*_sub_ of 60 °C. Scale bars: 1 mm.

The inkjet-printed millimeter-sized letters and the University of Leeds icon, which were composed of the InP QDs, demonstrated excellent printing resolution, showing potential for Cd-free QDs-based anticounterfeiting applications and large-scale manufacturing (Figure 6). These patterns exhibited low roughness, were free of coffee rings, and displayed uniform luminescence, aligning with the previous discussion.

## Conclusions

We successfully fabricated Cd-free QLEDs without coffee rings based on high-quality InP/ZnSe_*x*_S_1–*x*_/ZnS QDs via IJP. The ME was introduced to alleviate the CRE, including the solutal ME and thermal ME, which were achieved by adjusting the vol % of the ink solvents and heating the substrate during printing, respectively. In particular, the thermal ME was found to play an important role in balancing the capillary flow and overcoming the CRE. The real-time evaporation of ink-20 revealed the SJ mode at 20 and 40 °C and the SS mode at 60 °C, giving insight into the fundamental understanding of the coffee ring formation of fluids containing NPs with an extremely small size (<10 nm). Furthermore, increasing the ETL thickness reduced trap density in the ETL and protected the QD layer from oxygen and water, greatly enhancing the electrical performance of InP QLEDs. The electrical performance of inkjet-printed QLEDs was inferior to spin-coated analogues because of the quenching of QDs during printing in the ambient atmosphere. Additionally, the hole mobility of PVK is much less than the electron mobility of Zn_0.9_Mg_0.1_O, resulting in an unbalanced charge injection. To further improve the electrical performance of inkjet-printed InP QLEDs, future work can focus on printing and fabricating in an inert atmosphere to avoid the degradation of QDs by water and oxygen and optimizing the ink solvents, which are compatible with TFB.

## Experimental Section

### Materials

Indium chloride (InCl_3_, 99.99%) and zinc chloride (ZnCl_2_, 98+%) were purchased from Thermo Fisher Scientific. Selenium powder (Se, 99.9%), magnesium acetate tetrahydrate (Mg(acet)_2_ 4H_2_O), and dimethyl sulfoxide were purchased from Alfa Aesar. Isopropyl alcohol (IPA), acetone, and oleic acid (OA) were purchased from VWR Chemicals. Zinc stearate (Zn(St)_2_), tetramethylammonium hydroxide (TMAH, 97%), squalane (SQL), zinc acetate (Zn(acet)_2_), trioctylphosphine (TOP), octanethiol, oleylamine, tris(dimethylamino)phosphine ((DMA)_3_P, 97%), sulfur powder (S, 99.98%), zinc acetate dihydrate (Zn(acet)_2_ 2H_2_O), ethanolamine, poly(3,4-ethylenedioxythiophene) polystyrenesulfonate (3%–4%, PEDOT:PSS), polyvinylcarbazole (PVK), poly(9,9-dioctylfluorene-*alt*-*N*-[4 *s*-butylphenyl]diphenylamine) (TFB), hexane, octane, cyclohexylbenzene (CHB, 97+%), and decane (99%) were purchased from Sigma-Aldrich. ITO glass (S211) and epoxy were purchased from Ossila. Aluminum (Al) wire (99.5%) was purchased from Advent Research Materials.

### Synthesis of Multishelled InP/ZnSe_*x*_S_1–*x*_/ZnS QDs

To synthesize the InP core, 0.45 mmol InCl_3_ and 2.2 mmol ZnCl_2_ were dissolved in 6.0 mL of oleylamine and degassed at 120 °C for 1 h. Then, 0.35 mL of (DMA)_3_P was rapidly injected into the solution at 180 °C under a nitrogen atmosphere and reacted for 10 min. To grow the shell, the following solutions were sequentially injected into the core solution and reacted at increasing temperatures: 1.0 mL of Se-TOP (0.12 mol/L) at 200 °C, 4.0 mL of Zn(St)_2_-SQL (4.74 mol/L) at 220 °C, a mixture of 0.5 mL of Se-TOP and 1.0 mL of *S*-TOP (2 mol/L) at 240 °C, 4.0 mL of Zn(St)_2_-SQL at 260 °C, a mixture of 0.17 mL of Se-TOP and 2.0 mL of *S*-TOP at 280 °C, each for 30 min. 4.0 mL of Zn(St)_2_-SQL was then injected and reacted at 300 °C for 1 h. Subsequently, 5.0 mL of octanethiol was added and reacted at 190 °C for 1 h. Afterward, 3.0 mL of Zn(acet)_2_-OA solution (1.0 mol/L) was injected and reacted at 190 °C for 2 h before cooling to room temperature (RT). The resulting InP/ZnSe_*x*_S_1–*x*_/ZnS QDs were diluted with hexane and centrifuged at 15,000 RCF for 5 min. The supernatant was mixed with IPA (three times the volume of hexane), and the mixture was centrifuged again at 15,000 RCF for 5 min to obtain pellets. The cleaning process was repeated at least three times, and the purified QDs were dispersed in octane for characterization.

### Synthesis of Zn_0.9_Mg_0.1_O-EA Nanoparticles

The synthesis protocol of colloidal Zn_0.9_Mg_0.1_O nanoparticles (NPs) was based on a previously published solution precipitation method, with minor modification.^60^ Initially, 576.2 mg of Zn(acet)_2_ 2H_2_O and 80.4 mg of Mg(acet)_2_ 4H_2_O were dissolved in 30 mL of dimethyl sulfoxide at RT for 1 h. Meanwhile, 5.0 mmol of TMAH was dissolved in 10 mL of IPA and stirred at RT for 1 h. TMAH solution was then slowly injected into another solution and reacted at RT for 4 h until the mixed solution was clear. The Zn_0.9_Mg_0.1_O NPs were precipitated and purified with acetone by centrifugation at 10,000 RCF for 10 min and finally dispersed in IPA. Then, 0.2 wt % ethanolamine was added into the Zn_0.9_Mg_0.1_O NP dispersion to improve its dispersity and then sonicated in the water bath for 1 h; the final NPs were denoted as Zn_0.9_Mg_0.1_O-EA. The ratio of 0.2 wt % was calculated by the volume of ethanolamine divided by the net mass of Zn_0.9_Mg_0.1_O NPs in the dispersion.

### Fabrication of InP QLEDs

First, ITO-coated glass was sonicated in Decon 90 (3%), Milli-Q water, and IPA consecutively for 20 min. The ITO glass was then dried under N_2_ flow and exposed to UV–ozone for 5 min to enhance its surface energy. Before spin coating, PEDOT:PSS was diluted with Milli-Q water with a volume ratio of 2:3 and then diluted with IPA with a volume ratio of 4:1. Zn_0.9_Mg_0.1_O-EA and diluted PEDOT:PSS were filtered by the 0.2 and 0.45 μm PTFE filters, respectively. Next, PEDOT:PSS, PVK (10 mg/mL, dissolved in chlorobenzene), QDs (∼15 mg/mL, in octane), and Zn_0.9_Mg_0.1_O-EA (∼25 mg/mL, in IPA) were sequentially spin-coated at 4000, 4000, 2000, and 3000 rpm, respectively, and annealed at 150 °C, 200 °C, 80 °C, and 100 °C, respectively, for 10 min. Subsequently, a 100 nm-thick Al cathode was deposited using the thermal evaporator (Edwards 306) with a deposition rate of 0.2 nm/s under a pressure of 2 × 10^–6^ bar. The air exposure time of ETL was about 20 min for transferring the devices from the glovebox to the thermal evaporator. Finally, the device was sealed with encapsulation epoxy and covered by a coverslip under UV exposure for 15 min.

For inkjet-printed devices, most assembly processes are the same as those for spin-coated QLEDs except for the assembly of the QD layer. QDs were dispersed in a mixture of CHB and decane (97/3, 9/1, 8/2, and 2/8, v/v) with a density of 10 mg/mL and then infilled in a cartridge (DMC-11601) with 1 pL nozzles (a diameter of 12 μm). The QD inks were printed by the Fujifilm printer (DMP2850) with a platen temperature of 20 °C, 40 °C, and 60 °C. The drop spacing, firing frequency, and voltage for printing were 20 μm, 2 kHz, and 9.5 V, respectively. After printing, the QD films were annealed in the glovebox at 110 °C for 10 min.

### Characterization

The size distribution and selected area electron diffraction of InP/ZnSe_*x*_S_1–*x*_/ZnS QDs and Zn_0.9_Mg_0.1_O-EA NPs were measured by transmission electron microscopy (TEM, FEI Titan Cubed Themis 300 G2). The elemental ratio of Zn_0.9_Mg_0.1_O-EA NPs was characterized by energy-dispersive X-ray spectroscopy (EDX) accompanied by TEM. The steady-state fluorescence, time-resolved PL, and PLQY were tested by a fluorimeter with an integrated sphere (FLS980, Edinburgh Instrument). The PL peak maxima was indicated by the wavelength at which the PL intensity reaches a maximum after smoothing the PL spectrum. The average lifetime (τ_avg_) of QDs was described by
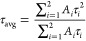
3*A*_*i*_ and τ _*i*_ are amplitudes and decay times of components. The absorbance was measured by a UV–vis spectrometer (Cary5000 UV–vis, Agilent), and the band gap was calculated from the absorbance curves, i.e., the Tauc plot of (α *h* ν)^2^ versus *h* ν, where α is the absorption coefficient (absorbance divided by the thickness of the sample), *h* is Planck’s constant, and ν is the frequency of light. The viscosity of QD inks was measured at 20 °C by a rheometer (MCR302, Anton Paar) with a CP-50 measuring tool at a shear rate of 10^3^ s^–1^. Surface tension and contact angle were tested by a tensiometer (OCA 15EC, DataPhysics Instruments GmbH) via pendent drop and sessile drop methods, respectively. The thickness of spin-coated films was characterized by the ellipsometer (M-2000, J A Woollam). The ellipsometry data were fitted by the Gen-Osc model (Gaussian for organic materials and Tauc-Lorentz for QDs), and the fitting quality was indicated by the mean square error. The density of QD inks was calculated by dividing the ink’s mass by its volume. The surface morphology of spin-coated and inkjet-printed films was measured by the tapping-mode atomic force microscopy (AFM, Multimode 8, Bruker) with a TESPA-V2 tip. The inkjet-printed patterns and top-view evaporation of QD inks were observed by the fluorescence microscope (E600, Nikon). The current density–voltage (*J*–*V*) characteristic was collected via a computer-controlled Keithley 2400 source meter. The electroluminescent (EL) spectra were recorded using a customized fiber-optic spectrometer (LS55, PerkinElmer) connected to the computer-controlled Keithley 2400 source meter. The luminance of QLEDs was measured with a digital luminance meter (TEN01070). EQE was calculated by the following equation^61^
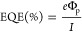
4where *e*, Φ_p_, and *I* represent the elementary charge, total photon flux, and current, respectively.

## Data Availability

The raw data associated with this article are available from https://doi.org/10.5518/1630.

## Supporting Information Available

Optical properties, size distribution, and selected area electron diffraction image of QDs and Zn_0.9_Mg_0.1_O NPs; stability of ink-20; thicknesses and mean square error of TFB films and PVK films before and after rinsing by the ink solvents; fluorescence microscopy images of inkjet-printed square patterns by applying various drop spacing; ejection of QD droplets under different printing voltages; contact angle of PEDOT:PSS inks with and without adding IPA varying with the UV–Ozone exposure time; AFM images and corresponding line profiles of the functional films; TEM images and EDS spectra of Zn_0.9_Mg_0.1_O NPs; luminance and EQE of inkjet-printed and spin-coated QLEDs; EL intensity and corresponding photographs of inkjet-printed InP QLEDs under various applied voltages; and EL spectra of InP QLEDs using TFB as the HTL (PDF)
