# Supplementary material for: Inkjet Printing of Cadmium-Free Quantum Dots-Based Electroluminescent Devices
Source: ACS Appl Mater Interfaces. 2025 Apr 3;17(15):22952–62. doi: 10.1021/acsami.5c01588 (PMC12012685; doi:10.1021/acsami.5c01588)
Supplement: Supplementary file 1 — am5c01588_si_001.pdf [file am5c01588_si_001.pdf]

Supporting Information

Inkjet Printing of Cadmium-Free Quantum Dots-Based Electroluminescent Devices

Min Fu,[a] Juan José Santaella,[b] Stephen D. Evans,[a] Kevin Critchley*[a]

[a] School of Physics and Astronomy, University of Leeds, Leeds, LS2 9JT, United Kingdom

[b] VALEO Lighting Systems, Department of Electronics, Martos, 23600, Spain

Email: k.critchley@leeds.ac.uk

The InP core showed a first excitation peak of 539 nm and an optical band gap of 2.13 eV (Figure S1a, b), but exhibited negligible photoluminescence (PL), resulting in a very low PL quantum yield (PLQY) of 1.6% was measured (Figure S1c and Table S1). This indicates many surface traps on the core surface. The optical bandgap was determined from the intercept between the wavelength axis and the tangent to the linear portion of the absorption band edge. The decay was clearly faster for the core-only sample compared to the core–shell–shell sample. None of the decay curves could be fitted to a single component exponential decay function which suggests, as expected, that there are competing pathways for relaxation. In this work, a two-component decay model was required to fit the fluorescence decay. Although there may be more processes, for example, processes with similar rates which we could not resolve, the two carrier relation processes in the QDs can be approximately assigned to a fast component (τ_1) that resulted from the carrier capture by defect states[1] and a slow component (τ_2) due to the band-edge electron-hole radiative recombination.[2] The two lifetime components were fixed, with only their amplitudes adjusted, as the decay pathways were assumed to remain consistent. However, the τ_1 of the core was found to be shorter than that of the core-shell QDs because surface trap states on the core were partially passivated after the shell coating. Notably, the contribution of τ_1 to the overall decay in core–shell QDs decreased significantly from 99.8% to 63.7%, but the τ_2% enhanced from 0.2% to 36.3%, indicating effective surface passivation. The fractional contribution of each lifetime component was calculated using:

$$\tau_i\ (\%) = \frac{A_i \tau_i}{\sum_{i=1}^{2} A_i \tau_i} \qquad (1)$$

The nonradiative decay rates (k_n) greatly decreased from 92.0 to 3.0 μs^{-1} after coating the shell, preventing the nonradiative decay that resulted from trap states and energy transfer in QDs. k_n was calculated based on the PLQY and τ_{avg}.[3]

$$\text{PLQY} = \frac{k_r}{k_t} = \frac{k_r}{k_n + k_r} = k_r \tau_{avg} \qquad (2)$$

Here, k_t and k_r represent total and radiative decay rates, respectively.

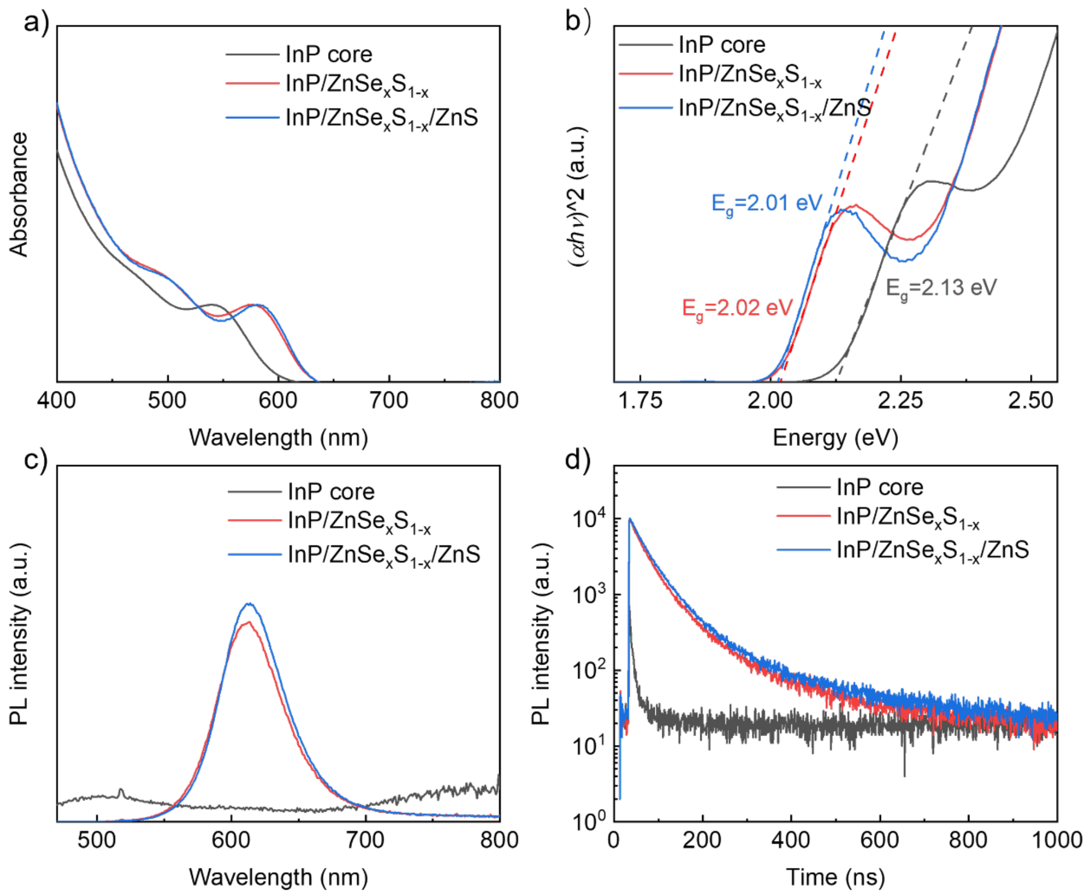

Figure S1. Optical properties of the synthesized InP QDs. a) UV-Vis spectrum, b) Tauc plot, c) steady-state PL, and d) time-resolved PL spectrum of InP core, InP/ZnSe$_x$S$_{1-x}$, and InP/ZnSe$_x$S$_{1-x}$/ZnS QDs.

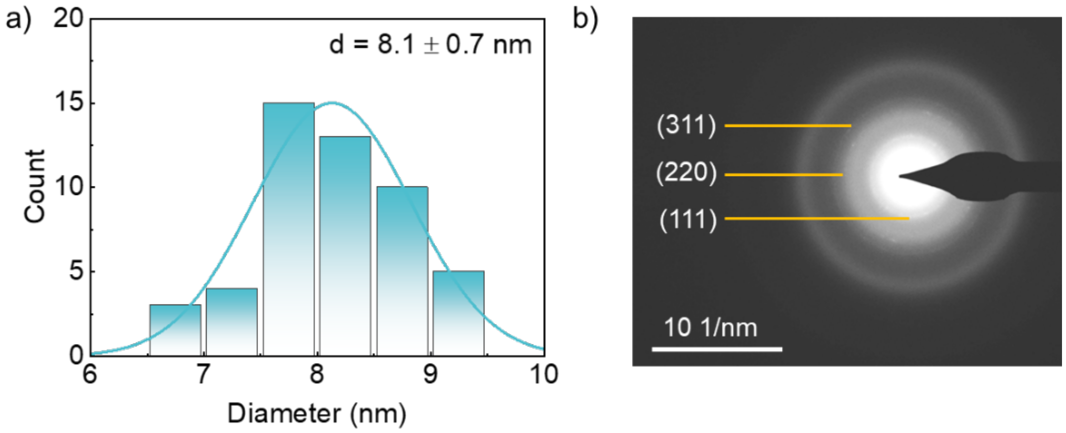

Figure S2. a) Size distribution and b) selected area electron diffraction image of InP/ZnSe$_x$S$_{1-x}$/ZnS QDs.

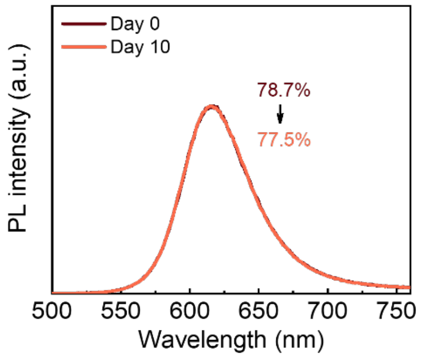

Figure S3. Stability of the ink-20 formulation stored in a glovebox for 10 days.

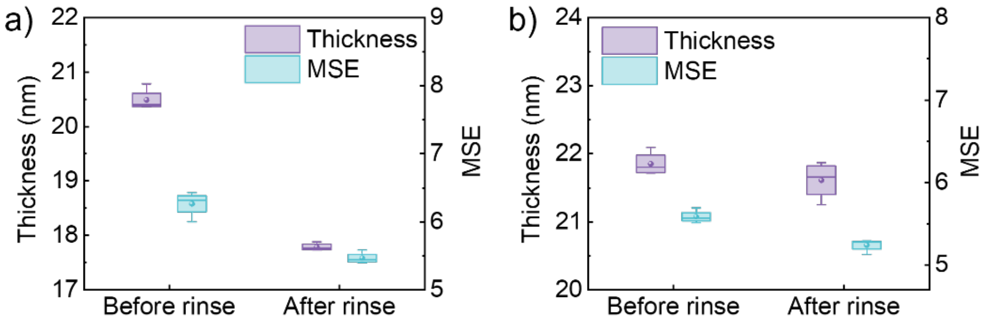

Figure S4. Thicknesses and mean square error (MSE) of a) TFB films and b) PVK films before and after rinsing by the ink solvents.

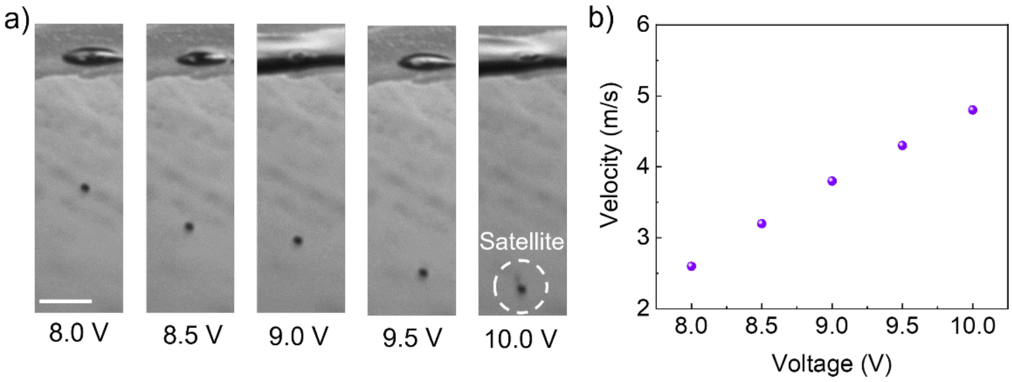

Figure S5. a) Images of the ejection of QD-ink droplets at 80 μs under various printing voltages. Scale bar: 100 μm. b) The velocity of QD droplets with respect to the printing voltage.

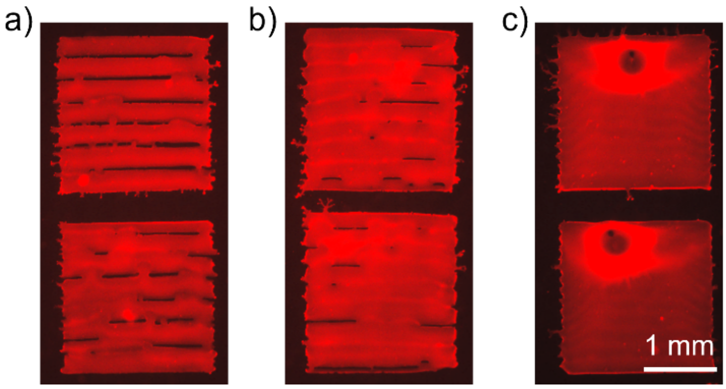

Figure S6. Fluorescence microscopy images of inkjet-printed square patterns by applying various drop spacing. a) 30 µm, b) 25 µm, c) 20 µm.

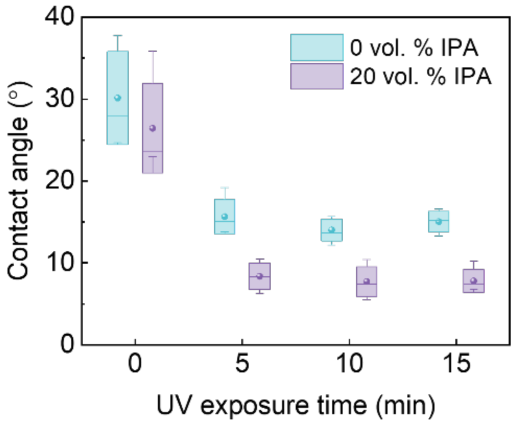

Figure S7. Contact angle of PEDOT:PSS inks with and without adding IPA varies with the UV-Ozone exposure time. The substrate is ITO glass.

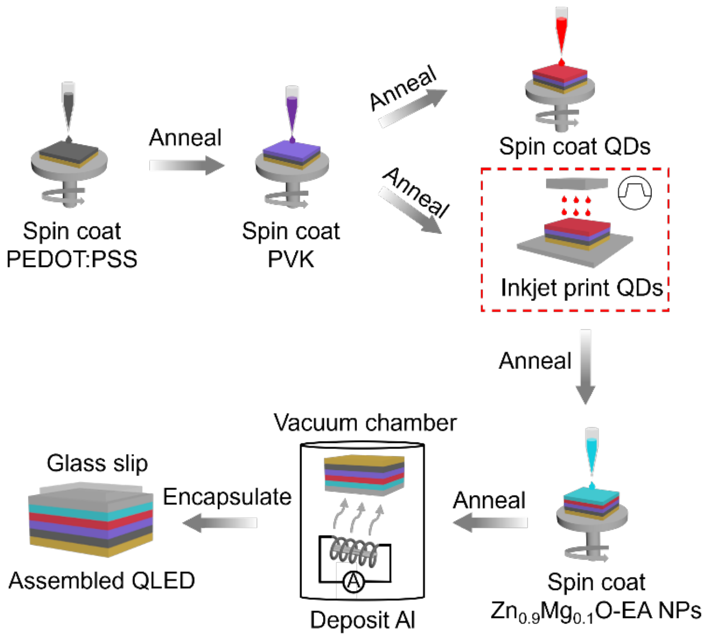

Scheme S1. A step-by-step assembly process of inkjet-printed and spin-coated InP QLEDs.

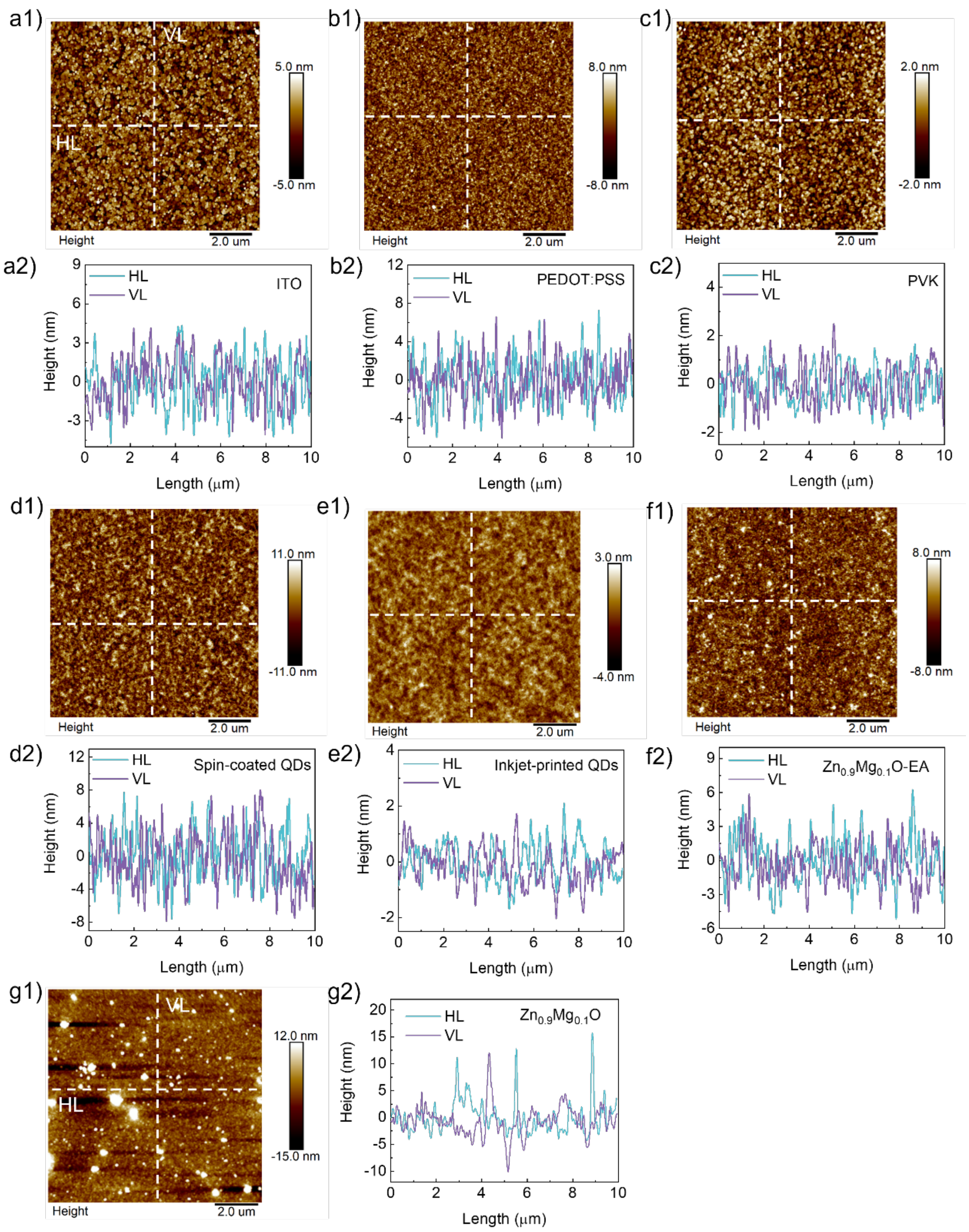

Figure S8. AFM images and corresponding line profiles of functional layers in the device stack. a1-a2) ITO, b1-b2) PEDOT:PSS, c1-c2) PVK, d1-d2) spin-coated QD films, e1-e2) inkjet-printed QD films, f1-f2) $Zn_{0.9}Mg_{0.1}O$-EA film, and g1-g2) $Zn_{0.9}Mg_{0.1}O$ film.

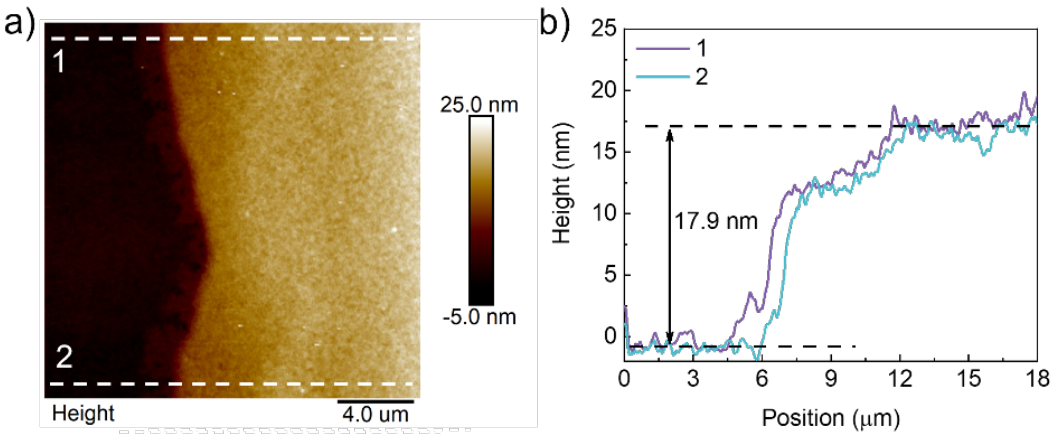

Figure S9. The AFM image of a) inkjet-printed QD films and b) corresponding thickness profiles.

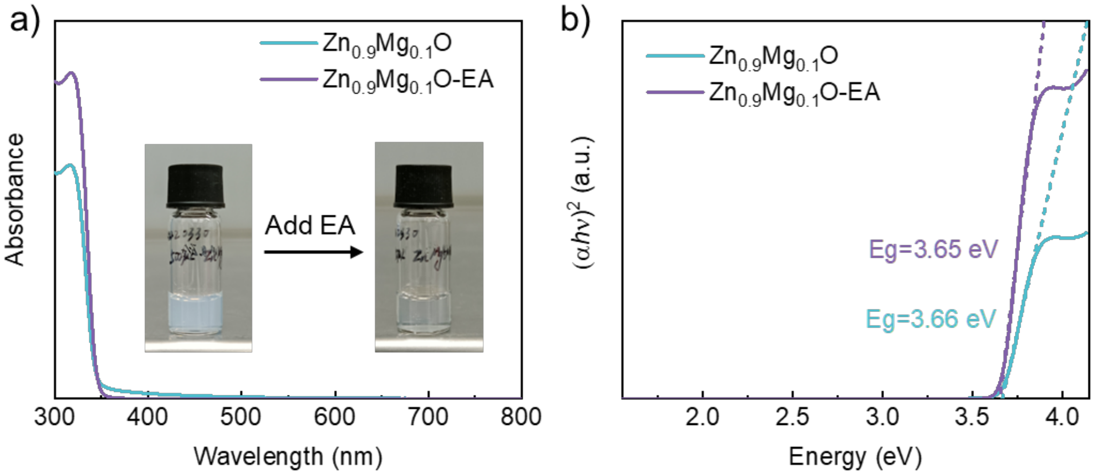

Figure S10. a) Absorption spectra and b) Tauc plots of $(\alpha h\nu)^2$ of $Zn_{0.9}Mg_{0.1}O$ and $Zn_{0.9}Mg_{0.1}O$-EA NPs. The insets are photos of $Zn_{0.9}Mg_{0.1}O$ NPs before and after adding ethanolamine.

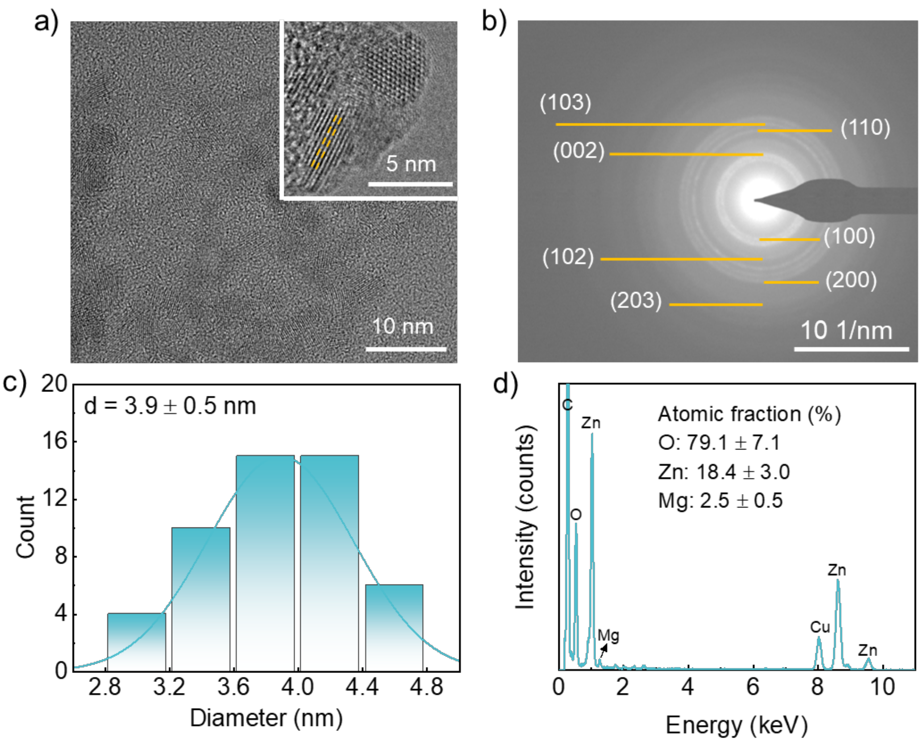

Figure S11. a) High-resolution TEM images and b) selected area electron diffraction images of $Zn_{0.9}Mg_{0.1}O$ NPs. An interplanar spacing of 2.73 Å corresponds to the (100) crystalline plane. c) Size distribution and d) EDS spectra of $Zn_{0.9}Mg_{0.1}O$ NPs.

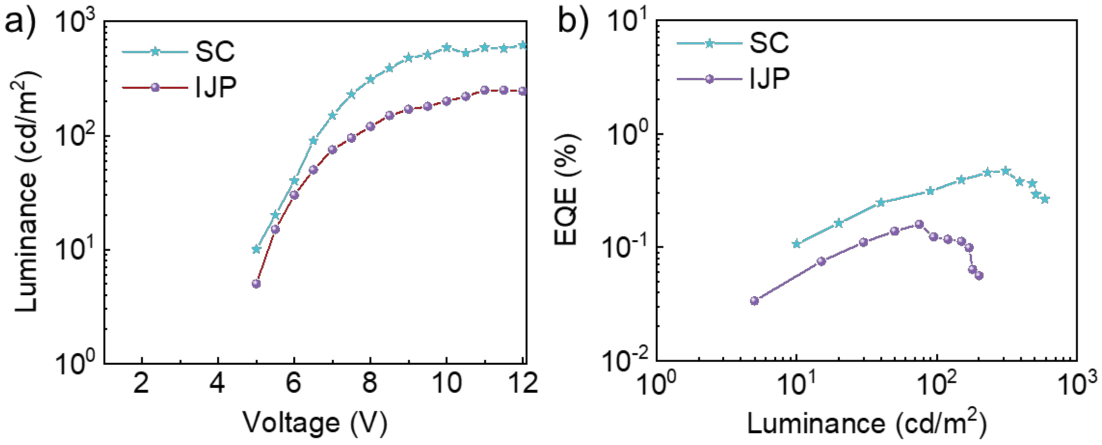

Figure S12. a) Luminance and b) EQE of inkjet-printed and spin-coated InP QLEDs.

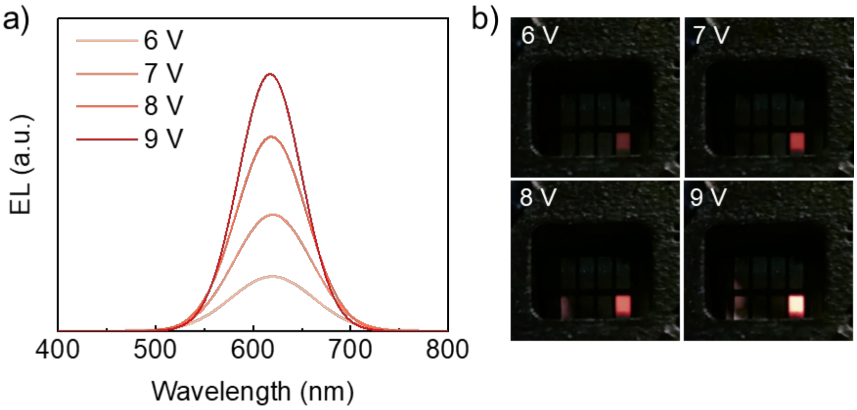

Figure S13. a) EL intensity and b) corresponding photographs of inkjet-printed InP QLEDs under various applied voltages. Each pixel is 2 mm × 2 mm.

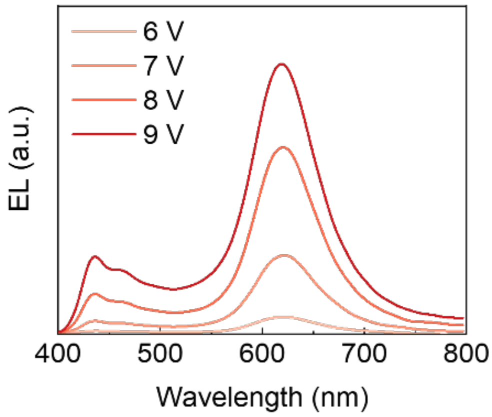

Figure S14. EL spectra of InP QLEDs using TFB as the HTL.

Table S1. Optical properties of InP core, InP/$ZnSe_xS_{1-x}$, and InP/$ZnSe_xS_{1-x}$/ZnS QDs.

	InP core	InP/$ZnSe_xS_{1-x}$	InP/$ZnSe_xS_{1-x}$/ZnS
First excitation peak (nm)	539	576	581
Emission peak (nm)	N.A.	613	614
FWHM (nm)	N.A.	58	56
Stokes shift (nm)	N.A.	37	33
Average lifetime (ns)	14.2 ± 2.3	65.6 ± 1.4	72.0 ± 2.3
PLQY (%)	1.6 ± 1	57.9 ± 0.4	80.8 ± 1.6
E_g (eV)	2.13	2.02	2.01

Table S2. PL lifetime components and nonradiative rates of InP core, InP/$ZnSe_xS_{1-x}$, and InP/$ZnSe_xS_{1-x}$/ZnS QDs.

	InP core	InP/$ZnSe_xS_{1-x}$	InP/$ZnSe_xS_{1-x}$/ZnS
A_1	36.7	8307.7	8803.5
τ_1 (ns)	10.5 (99.8%)	33.6 (68.0%)	33.6 (63.7%)
A_2	0.006	1140.7	1466.5
τ_2 (ns)	114.9 (0.2%)	114.9 (32.0%)	114.9 (36.3%)
τ_{avg} (ns)	10.7	59.6	63.1
χ^2	1.5	1.5	1.8
k_n (μs^{-1})	92.0	7.0	3.0

Table S3. A summary of the thickness and roughness of spin-coated PEDOT:PSS, PVK, QDs, and $Zn_{0.9}Mg_{0.1}O$ films measured by ellipsometry and AFM respectively.

Samples	Spin speed (r.p.m)	Ellipsometry		AFM
		Thickness (nm)	MSE	Roughness (R_{ms}, nm)
ITO	N.A.	101.1	5.2 ± 0.2	2.1 ± 0.1
PEDOT:PSS	4000	35 ± 1	6.3 ± 1.9	2.4 ± 0.2
PVK	4000	22 ± 1	2.1 ± 0.1	1.0 ± 0.1
QDs	2000	19 ± 1	1.9 ± 0.3	2.4 ± 0.4
$Zn_{0.9}Mg_{0.1}O$ (14 mg/mL)	4000	40 ± 1	3.3 ± 0.6	2.6 ± 0.2
$Zn_{0.9}Mg_{0.1}O$ (25 mg/mL)	5000	49 ± 1	6.9 ± 0.6	N.A.
	3000	61 ± 1	9.5 ± 0.2	N.A.

Reference

(1) Nguyen, A.; Jen-La Plante, I.; Ippen, C.; Ma, R.; Kelley, D. Extremely Slow Trap-Mediated Hole Relaxation in Room-Temperature InP/ZnSe/ZnS Quantum Dots. *J. Phys. Chem. C* **2021**, *125*, 4110-4118.
(2) Xu, Y.; Yan, L.; Fu, S.; Lv, Y. Shell thickness influence on the carrier dynamics of InP/ZnS QDs. *Chem. Phys. Impact* **2024**, *8*, 100579.
(3) Jiang, W.; Choi, Y.; Chae, H. Efficient green indium phosphide quantum dots with tris(dimethylamino)-phosphine phosphorus precursor for electroluminescent devices. *J. Mater. Sci. Mater. Electron.* **2021**, *32*, 4686-4694.
